# Differential effects of Fe^2+^ and Fe^3+^ on osteoblasts and the effects of 1,25(OH)_2_D_3_, deferiprone and extracellular calcium on osteoblast viability under iron-overloaded conditions

**DOI:** 10.1371/journal.pone.0234009

**Published:** 2020-05-29

**Authors:** Kornkamon Lertsuwan, Ketsaraporn Nammultriputtar, Supanan Nanthawuttiphan, Natnicha Tannop, Jarinthorn Teerapornpuntakit, Jirawan Thongbunchoo, Narattaphol Charoenphandhu

**Affiliations:** 1 Department of Biochemistry, Faculty of Science, Mahidol University, Bangkok, Thailand; 2 Center of Calcium and Bone Research (COCAB), Faculty of Science, Mahidol University, Bangkok, Thailand; 3 Department of Physiology, Faculty of Science, Mahidol University, Bangkok, Thailand; 4 Department of Physiology, Faculty of Medical Science, Naresuan University, Phitsanulok, Thailand; 5 Institute of Molecular Biosciences, Mahidol University, Nakhon Pathom, Thailand; 6 The Academy of Science, The Royal Society of Thailand, Dusit, Bangkok, Thailand; Università degli Studi della Campania, ITALY

## Abstract

One of the potential contributing factors for iron overload-induced osteoporosis is the iron toxicity on bone forming cells, osteoblasts. In this study, the comparative effects of Fe^3+^ and Fe^2+^ on osteoblast differentiation and mineralization were studied in UMR-106 osteoblast cells by using ferric ammonium citrate and ferrous ammonium sulfate as Fe^3+^ and Fe^2+^ donors, respectively. Effects of 1,25 dihydroxyvitamin D_3_ [1,25(OH)_2_D_3_] and iron chelator deferiprone on iron uptake ability of osteoblasts were examined, and the potential protective ability of 1,25(OH)_2_D_3_, deferiprone and extracellular calcium treatment in osteoblast cell survival under iron overload was also elucidated. The differential effects of Fe^3+^ and Fe^2+^ on reactive oxygen species (ROS) production in osteoblasts were also compared. Our results showed that both iron species suppressed alkaline phosphatase gene expression and mineralization with the stronger effects from Fe^3+^ than Fe^2+^. 1,25(OH)_2_D_3_ significantly increased the intracellular iron but minimally affected osteoblast cell survival under iron overload. Deferiprone markedly decreased intracellular iron in osteoblasts, but it could not recover iron-induced osteoblast cell death. Interestingly, extracellular calcium was able to rescue osteoblasts from iron-induced osteoblast cell death. Additionally, both iron species could induce ROS production and G0/G1 cell cycle arrest in osteoblasts with the stronger effects from Fe^3+^. In conclusions, Fe^3+^ and Fe^2+^ differentially compromised the osteoblast functions and viability, which can be alleviated by an increase in extracellular ionized calcium, but not 1,25(OH)_2_D_3_ or iron chelator deferiprone. This study has provided the invaluable information for therapeutic design targeting specific iron specie(s) in iron overload-induced osteoporosis. Moreover, an increase in extracellular calcium could be beneficial for this group of patients.

## Introduction

Iron overload could be a result from an increase in iron absorption, ineffective erythropoiesis and regular blood transfusion [1,2]. These conditions are commonly found in several diseases, e.g., β-thalassemia, hereditary hemochromatosis and sickle cell anemia [3–5]. Because the body has no active mechanism for effective iron excretion [2,6], excess iron often leads to iron toxicity in a number of organs, such as heart, liver and bone [2,7,8], the latter of which could lead to massive calcium loss, osteoporosis, pathological fracture and deformity. Thus, many patients with iron overload have been reported to exhibit signs of osteopenia or osteoporosis [9–11].

Two forms of ionized iron exist in the biological systems—i.e., ferrous (Fe^2+^) and ferric iron (Fe^3+^) [12]. Regarding intestinal absorption, free-ionized non-heme Fe^3+^ iron must be reduced to Fe^2+^ by ferric reductase duodenal cytochrome b (DcytB) before being transported into cells, mostly via divalent metal transporter (DMT)-1. Once inside the cells, iron is either stored in iron storage protein, ferritin or entering mitochondria to be used for heme and iron-sulfur cluster synthesis. Fe^2+^ is exported from the intestinal cells by ferroportin-1 and oxidized back to Fe^3+^ by copper-dependent ferroxidase hephaestin before binding to transferrin and circulated through the body [13–15]. Excess free iron in the cells could participate in redox reaction for reactive oxygen species (ROS) production through interconverting between Fe^3+^ and Fe^2+^, leading to organ damage and a number of diseases, e.g., fibrosis, liver injury, heart failure, diabetes mellitus, neurodegenerative diseases and bone loss [5,16,17].

Cellular iron metabolism in bone cells is largely unclear. For example, the principle route for osteoblast iron uptake is controversial, but it might be related to DMT1, some calcium channels and/or transferrin receptor-mediated endocytosis. Nevertheless, ROS from iron overload has been reported to inhibit osteoblast differentiation and stimulate osteoclast differentiation, so bone loss could be the consequence [18,19]. Previously, we reported that Fe^3+^ was a dominant iron specie that inhibited osteoblast by decreasing osteoblast survival, proliferation and activity [20]. Although it is known that iron overload can disturb bone homeostasis causing bone loss, it is not known whether Fe^3+^ and Fe^2+^ have similar effects on osteoblast differentiation. Moreover, the effects of deferiprone (DFP) as a potential therapeutic agent for osteoblast cell suppression under iron overload were also tested in this study.

Human bone marrow derived-mesenchymal stem cells (hBMSCs) can differentiate into multiple committed cell types, e.g., chondrocytes, cardiomyocytes and osteoblasts. Osteoblast differentiation is regulated epigenetically and genetically by different proteins, cytokines and miRNAs [21,22]. Previous studies showed that an inhibition of histone deacetylases (HDACs), especially HDAC2, could facilitate hBMSC osteogenic differentiation [22]. Moreover, bone morphogenetic protein (BMP)-2 and miR-29c-3p were also shown to regulate osteoblast differentiation through Wnt/β-catenin pathway [21,23].

Osteoblast differentiation is driven by the sequential expression of multiple proteins classified as early, intermediate and late osteoblast differentiation markers responsible for osteoblast proliferation, maturation and mineralization, respectively. *Runt*-related transcription factor (Runx)-2 is the principal transcription factor, which further regulates other early differentiation markers including osterix (OSX) and alkaline phosphatase (ALP). Later on, intermediate osteoblast differentiation markers including collagen type 1 and osteopontin are expressed during pre-osteoblast development into mature osteoblasts. Osteocalcin is then expressed marking the stage of mature osteoblasts [22,24]. However, the differential effects of Fe^3+^ and Fe^2+^ on osteoblast differentiation marker expression have never been studied or compared. Additionally, 1,25 dihydroxyvitamin D_3_ (1,25(OH)_2_D_3_)—a potent enhancer for intestinal calcium absorption—profoundly benefits bone homeostasis, in part by increasing calcium availability for bone formation and delaying bone resorption. Furthermore, 1,25(OH)_2_D_3_ was also shown to directly modulate osteoblast proliferation, differentiation and mineralization of osteoblasts (for reviews, please see [25–27]). It is possible that 1,25(OH)_2_D_3_ is able to help protect against adverse effects of iron overload on bone homeostasis. Our previous study illustrated that osteoblasts expressed similar iron transporters as found in enterocytes (e.g., the presence of DMT1 expression), and extracellular iron exposure enhanced iron transport into osteoblasts [20]. However, the effects of 1,25(OH)_2_D_3_ on iron uptake capacity of osteoblasts remained elusive, and the effects of 1,25(OH)_2_D_3_ and extracellular calcium on osteoblast viability under iron overload has never been elucidated.

Accordingly, this study is aimed to examine the effects of two different iron species, including Fe^3+^ and Fe^2+^, on osteoblast differentiation markers and osteoblast mineralization ability in UMR-106 cells by using ferric ammonium citrate (FAC) and ferrous ammonium sulfate (FAS) as Fe^3+^ and Fe^2+^ donors, respectively. As the known calcium absorption enhancer, effects of 1,25(OH)_2_D_3_ on iron uptake and cell survival of osteoblasts under iron overload with the two iron species were determined. Moreover, effects of extracellular calcium supplement on osteoblast cell survival under iron overload were also examined. Because deferiprone (DFP) has been used as an iron chelator to protect iron excess in iron overload patients [28,29], effects of DFP on iron uptake ability and osteoblast cell survival under iron overload with FAC and FAS were determined. Last but not least, the comparative effects of Fe^3+^ and Fe^2+^ on ROS production and cell cycle progression in osteoblasts under iron overload were elucidated.

Due to the ability to retain cell proliferation, several osteoblast models derived from osteosarcoma or stem cells were used. Several osteosarcoma-derived osteoblast cells were widely used to represent osteoblasts or cells committed to osteoblast linage, such as MG-63, Saos-2, U-2 OS and UMR-106 cell lines [30,31]. Therefore, this study provided the critical information for the development of treatment for iron overload-induced osteoporosis using specific therapeutic agents targeting specific iron specie(s) or mechanism underlies iron overload-induced osteoblast cell death in UMR-106 cells derived from rat osteosarcoma. UMR-106 cells have similar phenotypic characteristics to those found in primary osteoblasts, such as morphological appearance, high ALP activity, expression of vitamin D receptors and in vitro mineralization within 6 days after seeding [32–37].

## Materials and methods

### Cell culture and reagents

UMR-106 cells were obtained from American Type Culture Collection (ATCC no. CRL-1661). Cells were maintained and propagated at the Department of Physiology and Department of Biochemistry, Faculty of Science, Mahidol University, Thailand. UMR-106 cells were grown in Dulbecco’s modified Eagle’s medium (DMEM; Sigma Chemical CO., St. Louis, MO, USA) supplemented with 10% (v/v) fetal bovine serum (FBS; PAA Laboratories, Pasching, Austria) and 100 U mL^–1^ penicillin-streptomycin (Gibco, Grand Island, NY, USA). Cells were incubated at 37 °C with 5% CO_2_ and sub-cultured according to manufacturer’s instruction. The medium was replaced every other day.

Ferric ammonium citrate (FAC) and ferrous ammonium sulfate (FAS) were used as donors of Fe^3+^ and Fe^2+^, respectively (Sigma). Our previous study from viability assay (MTT assay) as well as cell proliferation assay (BrdU assay) at varied concentration of FAC and FAS showed that <30 μM of Fe^2+^ and Fe^3+^ affected neither viability nor proliferation of UMR-106 cells [20]. Thus, we used ≥30 μM Fe^2+^ and Fe^3+^ in the present study as we aimed to investigate the effects of iron on osteoblast viability. Moreover, we have shown that the short-term <300 μM Fe^2+^ and Fe^3+^ exposure did not significantly affect osteoblast cell viability [20]. Thus, 200 μM and 300 μM FAC and FAS were used in some experiments for a short period of time without affecting osteoblast viability. In this study, deferiprone (3-hydroxy-1,2-dimethyl-4(1H)-pyridone; DFP) (Sigma) was used as an iron chelator. Calcium chloride (CaCl_2_) (Merck, Kenilworth, NJ, USA) was used as extracellular calcium treatment.

### Quantitative real-time PCR (qRT-PCR)

Cells were plated at 4.2 × 10^5^ cells/well in 6-well plate (Corning, NY, USA). After seeding, cells were exposed to Fe^3+^ or Fe^2+^ iron donors (FAC and FAS) at 0, 100, 200 and 300 μM for 24, 48, and 72 h. Several studies showed that the expression alteration of osteoblast differentiation markers in different osteoblast cell lines including UMR-106 cells could be observed from 12 to 96 h of treatments, and the alteration of mRNA and protein expression was well correlated [38–42]. After incubation, the cells were collected by washing twice with phosphate buffered saline (PBS) solution and dissolved in TRIzol reagent (Invitrogen, Carlsbad, CA, USA) to extract total RNA. RNA was purified then measured the OD with NanoDrop-2000c spectrophotometer (Thermo Fisher Scientific, Waltham, MA, USA) at 260 and 280 nm. The ratio of which ranged between 1.8 and 2.0 was considered acceptable. Then, 1 μg of RNA was converted to complementary DNA (cDNA) with iScript cDNA synthesis kit (Bio-rad, Hercules, CA, USA) according to the manufacturer’s instruction.

The quantitative real-time PCR (qRT-PCR) was performed by QuantStudio 3D Digital PCR System with SsoFast EvaGreen Supermix (Bio-rad) for 40 cycles at 95 °C for 60 seconds, 55–60 °C annealing temperature (S1 Table) for 30 seconds and 72 °C for 30 seconds. Fold change values were calculated from the threshold cycles (C_T_) based on the standard ΔC_T_ method. Relative expression was expressed as the 2^–ΔΔCT^ method.

### Bone mineralization determination by alizarin red staining

Twenty-four hours after cell seeding, bone nodule formation of UMR-106 cells were induced by adding 50 mM β-glycerophosphate (Sigma) and 50 μg/mL l-ascorbate 2-phosphate (Sigma) with or without FAC or FAS (0–300 μM) for 6 days. It was previously reported that mineralization in UMR-106 cells occurred as early as 20 h after seeding, presumably due to high ALP activity [37,43], and alizarin red staining could be observed on day 4–5 [9,39]. The media was changed every other day. On day 6, cells were stained for calcium mineralization by alizarin red staining. The culture media was removed, and cells were gently washed with PBS for 3 times. Then, cells were fixed with 70% cold ethanol for 1 h at 4 °C. After fixing, cells were washed with deionized water 3 times. The water was completely removed, and cells were stained with 40 mM alizarin red S (Sigma) for 5–10 minutes on the shaker at room temperature. Alizarin red S was removed by washing 5 times with PBS or until the solution came out clear. Osteoblasts were inspected under a light microscope (Nikon, Melville, NY, USA), and the pictures were taken for further analysis. The total area of red nodule formation was quantified by Image J software (National Institutes of Health, USA).

### Flame atomic absorption spectrometry (FAAS)

Osteoblasts were seeded at 1 × 10^5^ cells/well in 12-well plates (Corning) and incubated for 24 h. UMR-106 cells were pre-treated with 10 nM 1,25(OH)_2_D_3_ (Cayman Chemical, Ann Arbor, MI, USA) without iron for 72 h. Then, cells were exposed to iron treatments (FAC or FAS) at 0, 100, 200 and 300 μM together with 10 nM 1,25(OH)_2_D_3_ or vehicle control for 24 h.

To determine the effects of deferiprone (DFP) on intracellular iron of osteoblasts under iron overload, after 1,25(OH)_2_D_3_ treatment mentioned above, cells were washed twice in cold PBS. Then, 100 μM DFP (Sigma) in PBS was added and incubated with slowly shaker at 4 °C for 30 minutes.

After the treatments, the solution was removed, and cells were washed again with PBS before gently scraped with cell scraper (Corning) in PBS. After centrifugation at 7,000 rpm for 15 minutes, the pellet was re-suspended and briefly sonicated in 250 μL of ultrapure water. Then, the samples were digested in 65% nitric acid (HNO_3_) and 30% hydrogen peroxide (H_2_O_2_) by Ethos UP MAXI-44 microwave digester (Milestone, CT, USA) as mentioned previously. After digestion, the samples were adjusted the volume to 25 mL with ultrapure water.

Intracellular iron was measured by FAAS. The system was calibrated with blank solution (2% HNO_3_), and the working standard in optimum range was used. Data analysis for intracellular iron concentration was normalized to protein concentration measured by bicinchoninic acid assay (BCA assay).

### Cell viability assay

To study the effects of 1,25(OH)_2_D_3_ on osteoblast cell viability under iron overload, UMR-106 cells were plated in 24-well plates at 2 × 10^4^ cells/well. Twenty-four hours after plating, cells were treated with 10 nM 1,25(OH)_2_D_3_ (Cayman Chemical, Ann Arbor, MI, USA) or 9:1 propylene glycol-ethanol as a vehicle control for 72 h. Then iron treatments (FAC or FAS) at 0, 100, 200 and 300 μM were introduced to the cells together with 10 nM 1,25(OH)_2_D_3_ or vehicle control for 24 h.

To study the effects of deferiprone (DFP) and CaCl_2_ on osteoblast cell viability under iron overload, the cells were plated at 1,000 cells/well on 96-well plates and allowed to attach to the plates for 24 h. After that, cells were treated with either FAC or FAS at 0, 30, 100 and 200 μM in the presence or absence of 100 μM DFP for 72 h. Similarly, the cells will be exposed to FAC or FAS at 0, 30, 100 and 200 μM with or without 1mM and 2.5 mM CaCl_2_ for 72 h.

At the end of the treatment period, cell viability was determined by MTT assay by adding 0.5 mg/mL thiazolyl blue tetrazolium bromide (MTT; Sigma) in culture media and incubating at 37 °C for 3 h. After incubation, MTT solvent (5% (w/v) Sodium dodecyl sulfate (SDS) (Vivantis Technologies Sdn. Bhd., Malaysia) in 50% (v/v) N, N-dimethylformamide (VWR international, LLC, OH, USA) in purified water was added in each well. The absorbance was measured with microplate reader (Metertech Inc., Taiwan) at 540 nm according to Riss et al. (2016) [44]. An absorbance was subtracted from culture medium background and normalized to control group (vehicle control without iron treatment). Then the absorbance was calculated as a percentage of cell viability.

### Cellular reactive oxygen species (ROS) assay

UMR-106 cells were seeded at 2.5 × 10^4^ cells/well in 96-black well plate (Corning). After 24 h, ROS production was measured by using DCFDA cellular ROS detection assay kit (Abcam, Cambridge, UK). The cells were washed with 1× buffer solution. Then, DCFDA solution was added 100 μL/well, and the cells were incubated for 45 minutes. After incubation, they were washed with 1× buffer again, then iron treatments were added at 0, 30, 100 and 200 μM and incubated for 6 h. ROS production was measured with fluorescence microplate reader (Spark^™^ 10M multimode microplate reader, Switzerland) at Ex/Em: 485/535 nm. Fold of ROS production was normalized to control groups.

### Cell cycle analysis

UMR-106 cells were seeded at 1.0 × 10^5^ cells/well in 6-well tissue culture plate (Corning). After 24 h, the cells were treated with 0, 30, 100, 200 and 300 μM of FAC and FAS (Sigma) for 72 h. The treatments were refreshed every day. Then, the cells were harvested by trypsinization and fixed in cold 70% ethanol overnight at –20 °C. Subsequently, each cell suspension was centrifuged at 800 rpm for 3 minutes and incubated with propidium iodide (PI) DNA staining solution (20 μg/mL) (Life Technologies, CA, USA) and 200 μg/mL DNAse-free RNAse A (Life Technologies, CA, USA) for 30 minutes at room temperature in the dark. Cell cycle distribution was analyzed using a FACScan flow cytometer (FACSCanto; BD Biosciences, USA).

### Statistical analysis

The results were expressed as Mean ± standard error of mean (SEM). The mean values were from at least 3 biological replicates with at least 3 internal repeats. The treatment groups were analyzed by the multiple comparison of one-way analysis of variance (ANOVA). The difference between pairs of means was analyzed by Tukey all pair comparison. The level of significance for all statistical tests was *P* < 0.05, and all data were analyzed by GraphPad Prism 5.0 (GraphPad Software Inc., San Diago, CA, USA).

## Results

### Ferric (Fe^3+^) and ferrous (Fe^2+^) altered osteoblast differentiation markers

**Fig 1:**
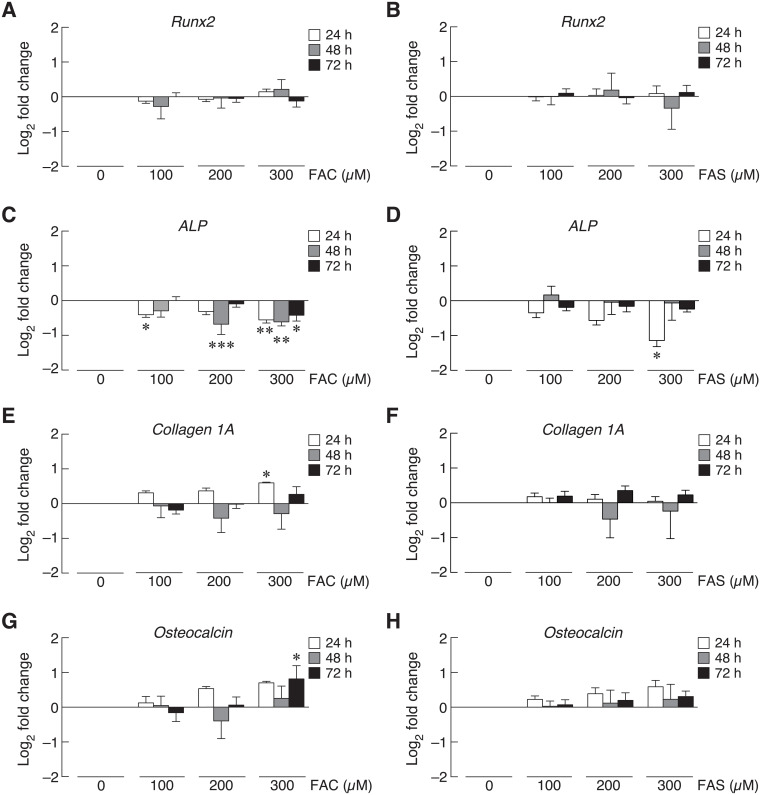
The expression of osteoblast differentiation markers in UMR-106 cells upon FAC and FAS exposure at 24, 48 and 72 h was determined by qRT-PCR. (A–B) Runx2, (C–D) alkaline phosphatase (ALP), (E–F) collagen I alpha (1A), and (G–H) osteocalcin expression upon FAC and FAS exposure. *P < 0.05, **P < 0.01, ***P < 0.001 as compared to control group (0 μM).

Osteoblast differentiation was determined by the expression of osteoblast differentiation factors, and iron overload has been reported to contribute to osteoblast differentiation impairment [9,45,46]. This study aimed to investigate the effects of two iron species, ferric (Fe^3+^) and ferrous (Fe^2+^), on the expression of osteoblast differentiation factors including runt-related transcription factor 2 (Runx2), alkaline phosphatase (ALP), collagen type 1A and osteocalcin by qRT-PCR. UMR-106 cells were exposed to ferric ammonium citrate (FAC) of ferrous ammonium sulfate (FAS) as Fe^3+^ and Fe^2+^iron donors, respectively. Twenty-four, 48 and 72 h treatments of Fe^3+^ or Fe^2+^ did not significantly change Runx2 mRNA expression level (Fig 1A and 1B). On the other hand, high concentration of both iron treatments significantly decreased ALP mRNA level at 24 h (Fig 1C and 1D). For 48- and 72-h exposure, the significant reduction of ALP mRNA level was found in only Fe^3+^ iron treatment but not Fe^2+^ (Fig 1C and 1D). Ferric treatment also had stronger effects on the expression of collagen type 1A at 24 h than Fe^2+^ iron. Interestingly, Fe^3+^ treatment at 200 and 300 μM triggered collagen type 1A mRNA expression (Fig 1E), but neither Fe^3+^ nor Fe^2+^ changed collagen type 1A mRNA level at 48 and 72 h (Fig 1E and 1F). Similar trend could be seen in the expression of osteocalcin. Only Fe^3+^ treatment at 300 μM for 72 h significantly increased osteocalcin mRNA level (Fig 1G); whereas, 24- and 48-h treatments of both iron species did not significantly affect osteocalcin expression (Fig 1G and 1H).

### Ferric (Fe^3+^) had higher deleterious effects on osteoblast mineralization than ferrous (Fe^2+^)

**Fig 2:**
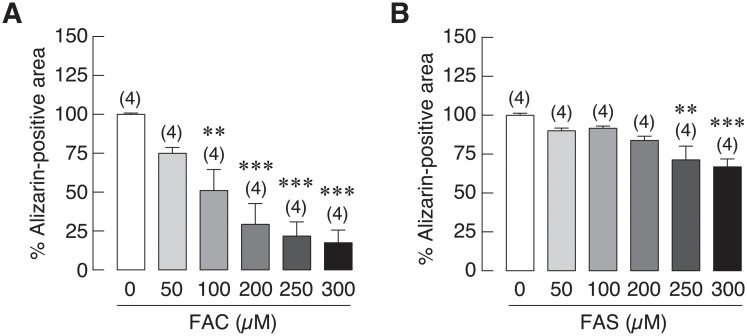
Quantified data from alizarin red S staining depicting calcium deposition of UMR-106 cells under iron overload with (A) FAC and (B) FAS. Both FAC and FAS significantly decreased osteoblast calcium deposition. **P < 0.01, ***P < 0.001 as compared to control group (0 μM).

Previous experiments showed that both iron forms could suppress the activity and expression of alkaline phosphatase (ALP), which is the key marker for bone mineralization ([20] and Fig 1C and 1D). Therefore, this experiment was hypothesized that iron overload from both iron species could also impair bone mineralization. Osteoblasts (UMR-106 cells) were incubated with 50 mM β-glycerophosphate and 50 μg/mL of l-ascorbate 2-phosphate to induce bone nodule formation in the presence or absence of Fe^3+^ or Fe^2+^ treatments for 6 days. After that, alizarin red staining was used to determine calcium deposition referred to bone mineralization after treatment either with Fe^3+^ or Fe^2+^. Quantified data showed that both Fe^3+^ and Fe^2+^ treatments significantly reduced the mineralization of bone nodule in a dose-dependent manner (Fig 2). Fe^3+^ significantly reduced osteoblast mineralization down to 51.04%, 29.27%, 21.79% and 17.49% at 100, 200, 250 and 300 μM, respectively (Fig 2A). On the other hand, osteoblast mineralization was slightly decreased to 71.29% and 66.79% in osteoblasts treated with 250 and 300 μM of Fe^2+^ (Fig 2B). Fe^3+^ treatment had stronger inhibitory effects on bone mineralization than Fe^2+^. This study confirmed that both forms of iron impaired bone mineralization, and the more drastic effects were observed in ferric treatments (S1 Fig).

### 1,25(OH)_2_D_3_ enhanced iron uptake in osteoblasts

**Fig 3:**
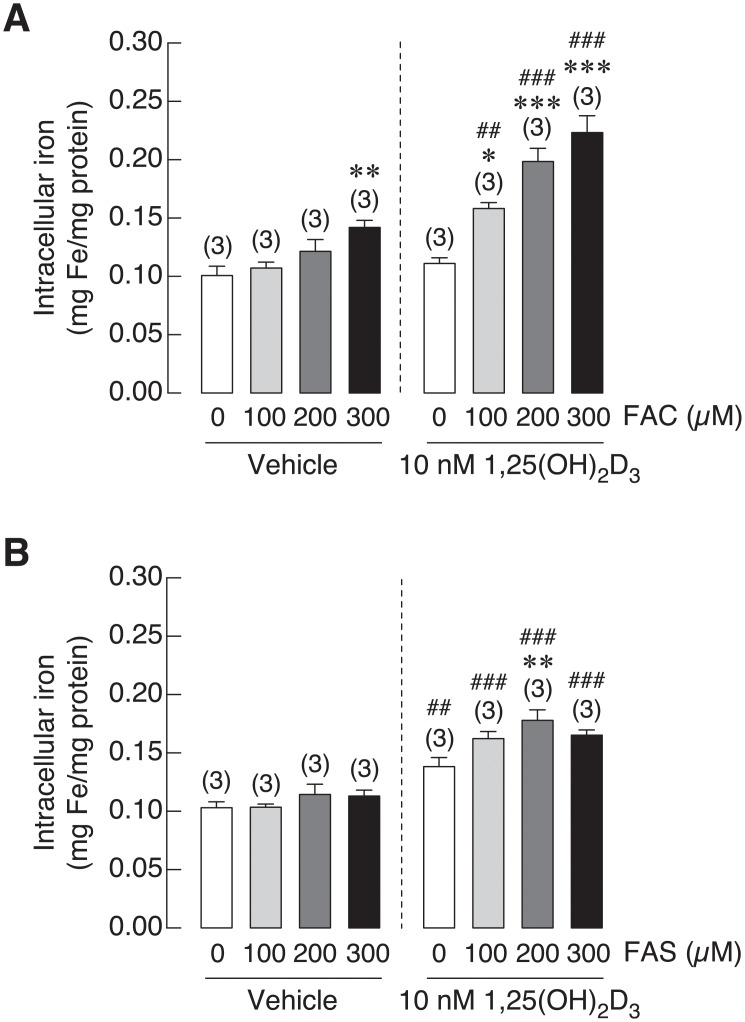
Intracellular iron was measured in UMR-106 cells exposed to (A) FAC and (B) FAS in the presence of 1,25(OH)_2_D_3_ or vehicle control. 1,25(OH)_2_D_3_ significantly induced intracellular iron in osteoblasts treated with FAC and FAS. *P < 0.05, **P < 0.01 ***P < 0.001 as compared to control group (0 μM) with vehicle control; ^##^*P* < 0.01, ^###^*P* < 0.001 as compared to the same dose of iron between vehicle and 1,25(OH)_2_D_3_ treatment.

1,25(OH)_2_D_3_ is an essential hormone for bone formation by inducing intestinal calcium transport. Interestingly, vitamin D deficiency and hypocalcemia were found in thalassemic patients with iron overload condition [47,48]. Therefore, 1,25(OH)_2_D_3_ might be beneficial for bone homeostasis of these patients. In addition, osteoblasts were also shown to express similar set of iron transporters found on enterocytes [20,49]. Accordingly, the relationship between 1,25(OH)_2_D_3_ and iron uptake in osteoblast is still needed to be uncovered. This experiment aimed to determine the effects of 1,25(OH)_2_D_3_ on iron uptake into osteoblasts upon Fe^3+^ and Fe^2+^ exposure. After treatment, intracellular iron concentration in osteoblast UMR106 cells were measured by flame atomic absorption spectrometry (FAAS). 1,25(OH)_2_D_3_ significantly increased intracellular iron concentration in osteoblasts treated with FAC and FAS as compared to vehicle treated groups. As shown in Fig 3A, intracellular iron increased in 1,25(OH)_2_D_3_ treated osteoblasts from 0.11 to 0.16 mg/mg proteins in cells treated with 100 μM FAC, from 0.12 to 0.20 mg/mg proteins with 200 μM FAC and from 0.14 to 0.22 mg/mg proteins with 300 μM FAC exposure. Likewise, treatment with 1,25(OH)_2_D_3_ also facilitated iron uptake into osteoblast treated with Fe^2+^ iron more than vehicle treated groups at the comparable doses of Fe^2+^ iron treatment. As shown in Fig 3B, intracellular iron increased from 0.10 to 0.16 mg/mg proteins with 100 μM FAS, from 0.11 to 0.18 mg/mg proteins with 200 μM FAS and from 0.11 to 0.17 mg/mg proteins with 300 μM FAS treatments with the presence of 1,25(OH)_2_D_3_. Our results showed that 1,25(OH)_2_D_3_ facilitated iron uptake of both iron species into osteoblasts, and we also found that Fe^3+^ was still a prefer form that was transported into osteoblasts.

### 1,25(OH)_2_D_3_ minimally affected iron-induced osteoblast cell death

**Fig 4:**
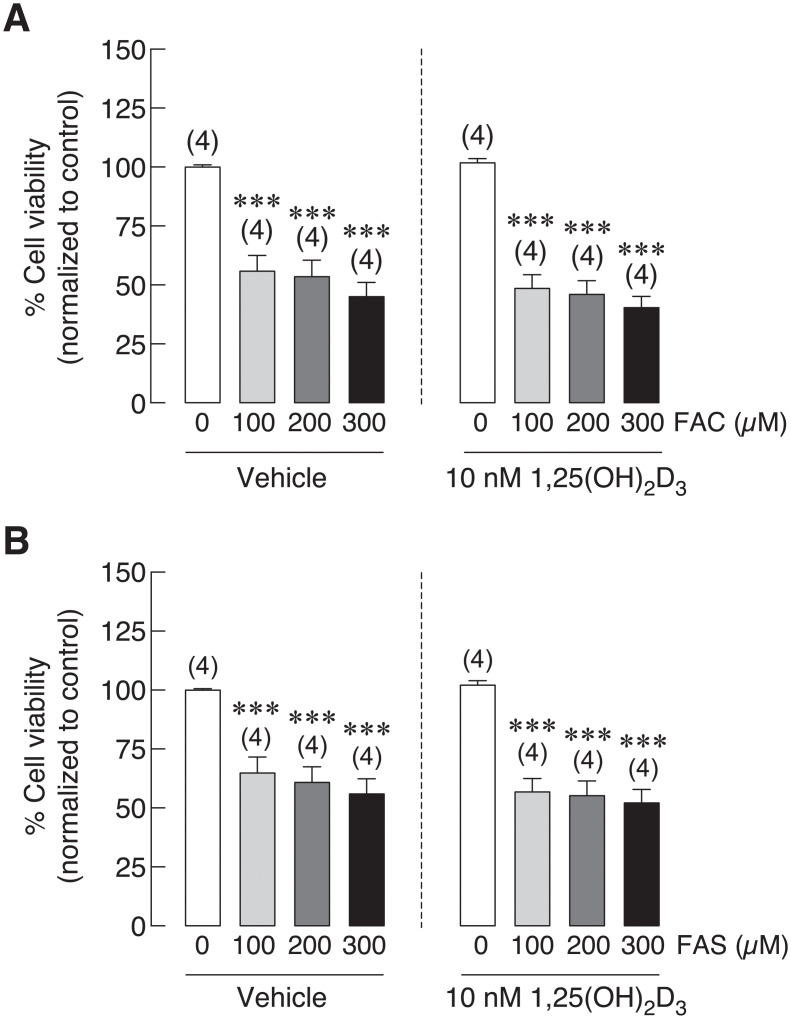
UMR-106 osteoblast cell viability was tested under the presence of (A) FAC or (B) FAS together with vehicle control or 1,25(OH)_2_D_3_. 1,25(OH)_2_D_3_ did not significantly affect osteoblast cell viability under iron overload with FAC and FAS. ***P < 0.001 as compared to control group (0 μM) with vehicle control.

Previous experiment showed that 1,25(OH)_2_D_3_ could enhance iron uptake leading to elevated intracellular iron in osteoblasts (Fig 3). To verify the effects of 1,25(OH)_2_D_3_ on osteoblast viability in the presence of iron, UMR-106 cells were pre-treated with 1,25(OH)_2_D_3_ for 72 h, then 1,25(OH)_2_D_3_ coupled with Fe^3+^ (FAC) or Fe^2+^ (FAS) for 24 h. Osteoblast viability was compared between vehicle and 1,25(OH)_2_D_3_-treated cells with the same concentration of Fe^3+^ and Fe^2+^ treatments. As shown in Fig 4A, 1,25(OH)_2_D_3_ treatment led to decreased osteoblast viability from 55.79% to 48.49% at 100 μM, from 53.54% to 45.98% in 200 μM and from 45.01% to 40.35% in 300 μM of FAC treated groups. However, no statistically significant difference was found. Similar effects were also shown in FAS treated groups. Osteoblast viability was decreased from 64.88% to 56.75% in 100 μM, from 60.86% to 55.25% in 200 μM and from 55.92% to 52.17% in 300 μM of FAS treated groups in the presence of 1,25(OH)_2_D_3_ (Fig 4B). Even though the further reduction in osteoblast viability was noted in osteoblasts treated with iron together with 1,25(OH)_2_D_3_, none of these results showed statistically different between the vehicle control and 1,25(OH)_2_D_3_ treatments.

### Deferiprone diminished 1,25(OH)_2_D_3_-induced intracellular iron in osteoblasts

**Fig 5:**
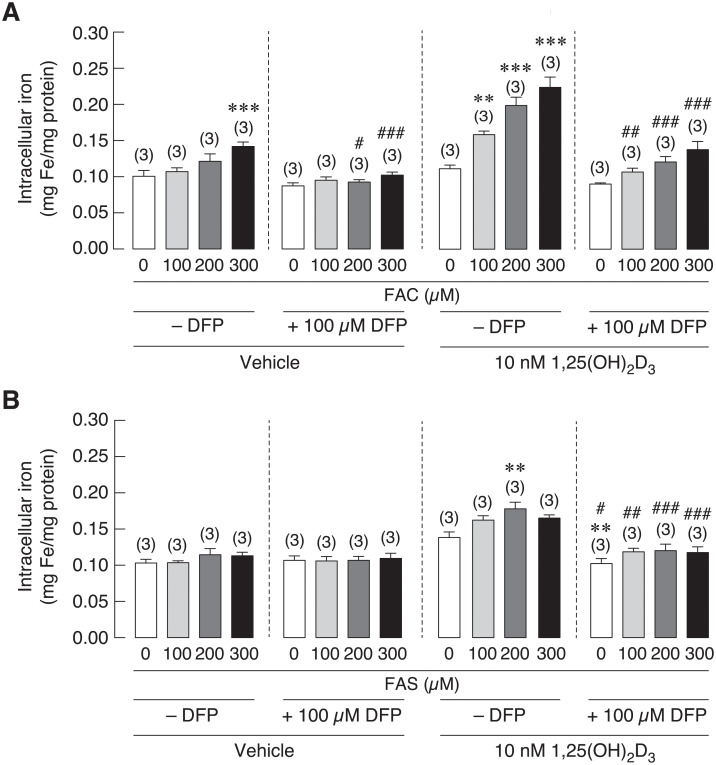
Intracellular iron in osteoblast UMR-106 cells after treated with (A) FAC and (B) FAS under the stimulation of vehicle control or 1,25(OH)_2_D_3_ in the presence or absence of deferiprone (DFP). DFP significantly reduced intracellular iron in osteoblasts treated with FAC and FAS. The more profound reduction was observed in the groups under 1,25(OH)_2_D_3_ stimulation. **P < 0.01 ***P < 0.001 as compared to control group (0 μM) without DFP. ^#^*P* < 0.05, ^##^*P* < 0.01, ^###^*P* < 0.001 as compared to the same iron concentration.

To further verify whether intracellular iron level relates to iron-induced osteoblast cell death under iron overload or not, effects of iron chelator on intracellular iron in osteoblasts under iron overload with FAC and FAS were tested. Deferiprone (DFP) was an iron chelator used to protect the excess iron in iron overload patients [28,29]. Therefore, this experiment aimed to study effects of DFP on intracellular iron of osteoblasts under iron overload with or without 1,25(OH)_2_D_3_ stimulation. After cells were exposed to vehicle control or 1,25(OH)_2_D_3_ and iron treatments, osteoblasts were incubated with DFP prior to intracellular iron measurement by FAAS. Overall, intracellular iron in osteoblast UMR-106 cells was decreased after the incubation with 100 μM deferiprone (Fig 5). In the absence of 1,25(OH)_2_D_3_, only high concentration of FAC at 300 μM could significantly raise intracellular iron in osteoblasts. No significant change was observed in other FAC or FAS treated groups; hence, the effect of DFP on the level of intracellular iron in these groups was not observed (Fig 5A). However, intracellular iron level in osteoblasts treated with 300 μM FAC was decreased significantly from 0.14 mg/mg proteins to 0.10 mg/mg proteins in the presence of DFP (Fig 5A). On the other hand, DFP notably lowered intracellular iron under 1,25(OH)_2_D_3_ stimulation from 0.11 to 0.09 mg/mg proteins in control groups, from 0.16 to 0.11 mg/mg proteins in 100 μM, from 0.20 to 0.12 mg/mg proteins in 200 μM and from 0.22 to 0.14 mg/mg proteins in 300 μM FAC treated groups (Fig 5B). Similarly, intracellular iron in FAS together with 1,25(OH)_2_D_3_ treated osteoblasts also decreased from 0.14 to 0.10 mg/mg proteins in control groups, from 0.16 to 0.12 mg/mg proteins in 100 μM, from 0.18 to 0.12 mg/mg proteins in 200 μM and from 0.17 to 0.12 mg/mg proteins in 300 μM FAS treated osteoblasts (Fig 5B**)**. These results demonstrated that iron chelator, DFP, could reduce 1,25(OH)_2_D_3_-induced intracellular iron in osteoblasts from both iron exposure.

### Deferiprone could not recover iron-induced osteoblast cell death

**Fig 6:**
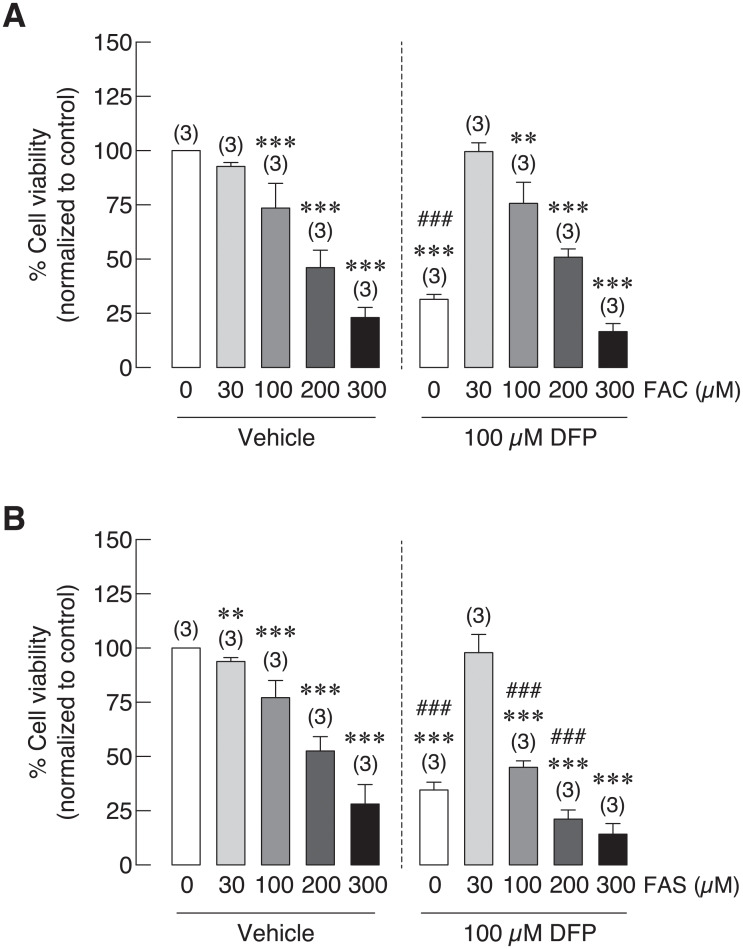
Osteoblast cell viability under iron overload from (A) FAC and (B) FAS in the presence of deferiprone (DFP). DFP could not recover iron-induced osteoblast cell death in both FAC and FAS treated groups. **P < 0.01 ***P < 0.001 as compared to control group (0 μM) without DFP. ^###^*P* < 0.001 as compared to the same iron concentration.

As previous experiment showed that deferiprone (DFP) could reduce intracellular iron in osteoblasts treated with FAC and FAS with and without 1,25(OH)_2_D_3_ (Fig 5). Effects of DFP on osteoblast cell survival under iron overload, especially those under 1,25(OH)_2_D_3_ stimulation were investigated. As shown in Fig 6A, FAC significantly reduced osteoblast cell survival in a dose-dependent manner both in vehicle control and DFP treated groups. While osteoblast cell survival was slightly improved in DFP treated groups: from 92.75% to 99.63%, from 73.54% to 75.66%, from 46.09% to 50.94% in osteoblasts treated with 30 μM, 100 μM and 200 μM FAC, respectively, none of these changes showed statistical significance. This effect could not be seen in osteoblasts treated with 300 μM FAC (Fig 6A). Similarly, dose-dependent inhibitory effects were also seen in FAS treated osteoblasts. However, a nonsignificant improvement of osteoblast cell survival was only seen in osteoblasts cells treated with 30 μM FAS from 93.83% in vehicle control to 97.89% in DFP treated group. It is worth to note that long-term exposure of DFP also significantly reduced osteoblast cell survival by itself. Thereby, the further reduction in osteoblasts exposed to iron together with DFP was also observed (Fig 6A and 6B).

### Extracellular calcium treatment recovered iron-induced osteoblast cell death

**Fig 7:**
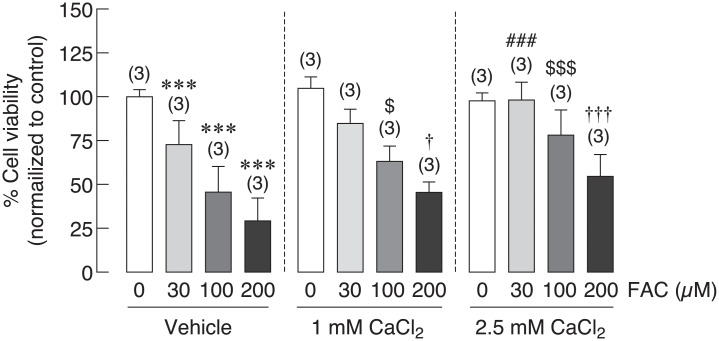
Effects of extracellular CaCl_2_ on UMR-106 osteoblast cell survival under iron overload with FAC were illustrated. Osteoblast cell viability was significantly improved ***P < 0.001 as compared to control group (0 μM) with vehicle control; ^###^*P* < 0.001 as compared to 30 μM FAC in vehicle control group; ^$^*P* < 0.05, ^$$$^*P* < 0.001 as compared to 100 μM FAC in vehicle control group; ^†^*P* < 0.05, ^†††^*P* < 0.001 as compared to 200 μM FAC in vehicle control group.

Hypocalcemia has been reported in thalassemia, which could worsen thalassemia and iron overload-induced osteoporosis [50–52]. Giving that calcium supplement could be one of the therapeutic agents for thalassemia and iron overload-induced osteoporosis, the direct effects of extracellular calcium on osteoblast cell viability under iron overload have not been elucidated. In this experiment, osteoblast UMR-106 cell viability was determined in osteoblasts treated with FAC at 0, 30, 100 and 200 μM in the presence or absence of CaCl_2_. Similar to results from previous experiments, this experiment also showed that FAC significantly reduced osteoblast cell viability in dose dependent manner. When extracellular calcium in a form of CaCl_2_ was applied, deleterious effect of ferric on osteoblast was subsided in a dose-dependent manner of CaCl_2_ (Fig 7). CaCl_2_ at 1 mM was able to rescue osteoblasts from FAC-induced osteoblast cell death by 17.63% and 16.31% in 100 and 200 μM FAC, respectively. More profound effects were shown in a higher concentration of CaCl_2_. Our results showed that osteoblast cell death was recovered by 25.38%, 32.61% and 25.43% in 30, 100 and 200 μM FAC treated groups in the presence of 2.5 mM CaCl_2_ (Fig 7).

### Both Fe^3+^ and Fe^2+^ induced cellular reactive oxygen species (ROS) production in osteoblasts

**Fig 8:**
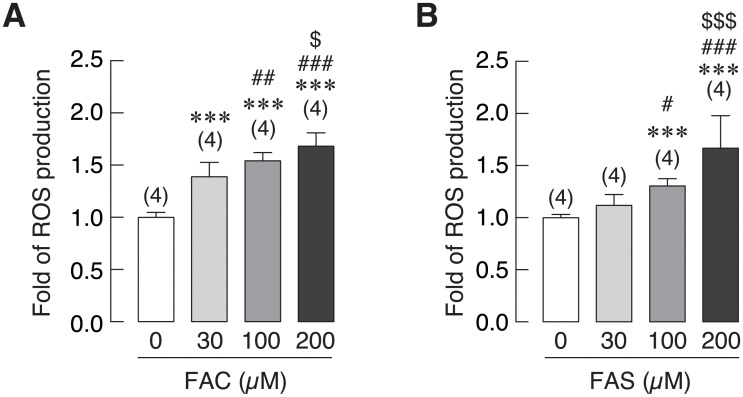
Intracellular reactive oxygen species (ROS) production in osteoblast cells treated with (A) FAC and (B) FAS was evaluated. Both FAC and FAS significantly increased ROS production in osteoblasts. ***P < 0.001 as compared to control group (0 μM); ^#^*P* < 0.05, ^##^*P* < 0.01, ^###^*P* < 0.001 as compared to 30 μM of iron; ^$^*P* < 0.05, ^$$$^*P* < 0.001, as compared to 100 μM of iron.

Reactive oxygen species (ROS) were shown to be associated with iron toxicity in several organs [53–55]. In this experiment, cellular ROS production in osteoblasts treated with FAC and FAS was measured to investigate whether this activity was related to iron-induced osteoblast cell death by both iron species. As shown in Fig 8A, FAC at 30, 100, and 200 μM significantly increased osteoblast ROS production to 1.39, 1.54 and 1.68-fold, respectively as compared to the control group (0 μM). In the same way, levels of cellular ROS in osteoblasts exposed to FAS was also increased significantly to 1.30 and 1.67-fold in 100 and 200 μM FAS treated groups as compared to control (Fig 8B). Hence, ROS level was increased in osteoblasts treated with both FAC and FAS in a dose-dependent manner.

### Fe^3+^ and Fe^2+^ caused G0/G1 cell cycle arrest in osteoblasts

**Fig 9:**
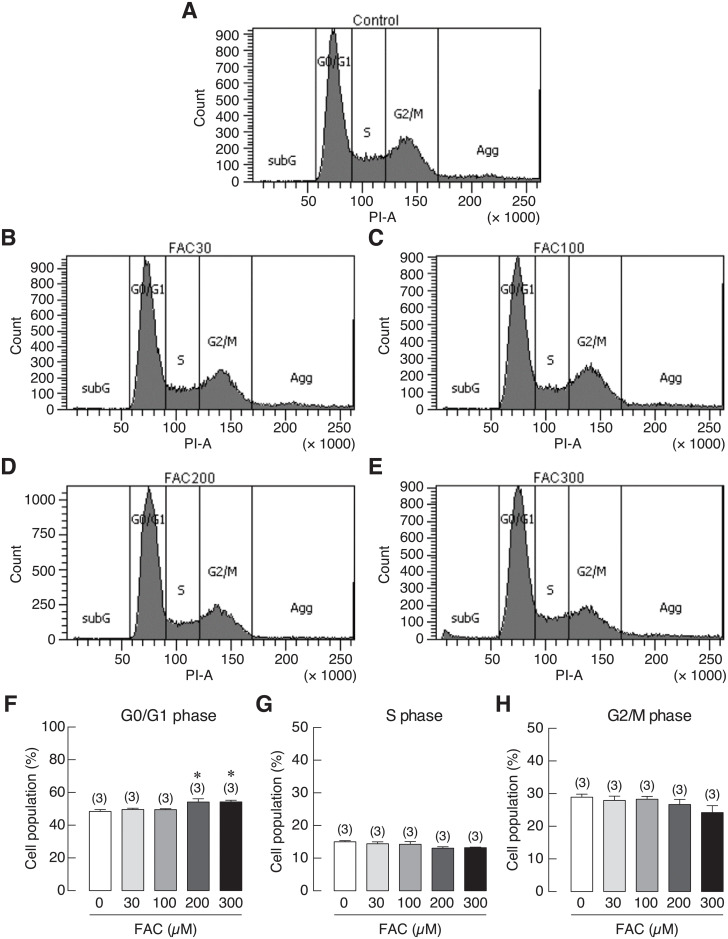
Cell cycle distribution of UMR-106 cells treated with 30, 100, 200 or 300 μM FAC. (A–E) Representative figures of cell cycle distribution as determined by flow cytometry. Quantified data showing percent cell distribution in G0/G1 phases (F), S phase (G) and G2/M phases (H). *P < 0.05 as compared to control group (0 μM); PI, propidium iodide.

**Fig 10:**
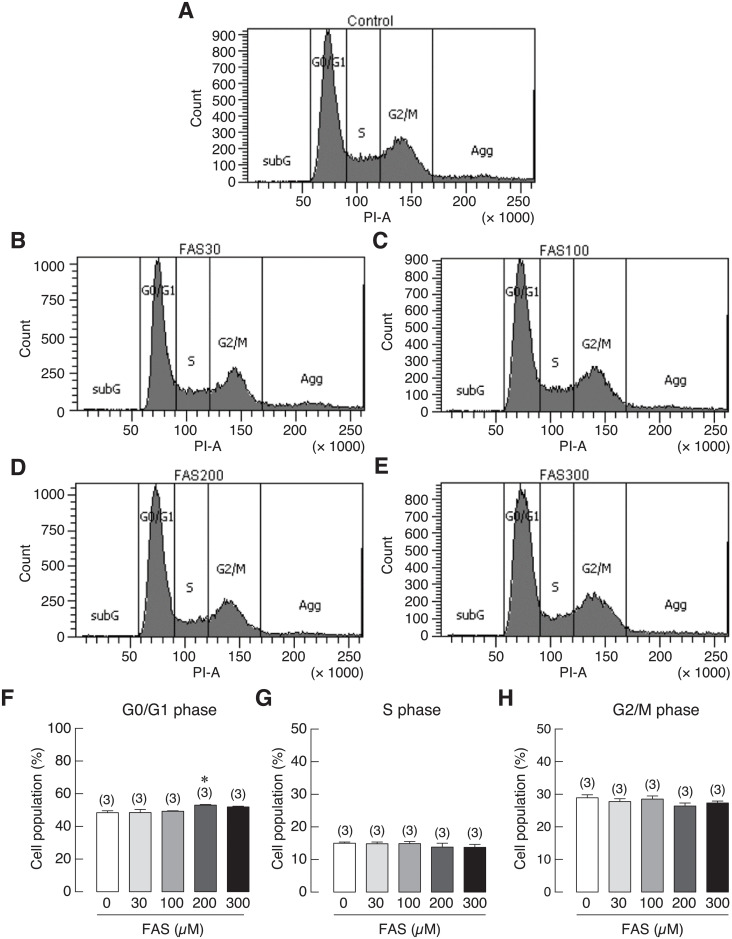
Cell cycle distribution of UMR-106 cells treated with 30, 100, 200 or 300 μM FAS. (A–E) Representative figures of cell cycle distribution as determined by flow cytometry. Quantified data showing percent cell distribution in G0/G1 phases (F), S phase (G) and G2/M phases (H). *P < 0.05 as compared to control group (0 μM); PI, propidium iodide.

To investigate the involvement of iron-induced cell growth inhibition in osteoblasts, cell cycle distribution of iron-treated UMR-106 cells was analyzed by flow cytometry. UMR-106 cells were treated with 0 (control), 30, 100, 200 and 300 μM of FAC or FAS for 72 h. The results showed that the percentage of cell population in the G0/G1 phase was significantly increased in 200 and 300 μM FAC-treated groups as compared to the corresponding control (Fig 9F). Similar results could be seen in osteoblasts treated with 200 μM FAS (Fig 10F). However, no significant change was observed in the S and G2/M phases. Our results thus suggested that FAC and FAS could induce cell cycle arrest in G0/G1 phase, resulting in cell growth inhibition in osteoblasts.

## Discussion

Osteoporosis in thalassemia and other iron overload-related diseases has been documented by many studies as reviewed in [45,56,57]. Interestingly, the information about the direct effects of iron on osteoblasts is still limited. As the two biological available forms of iron, our previous study was the first study to compare the direct deleterious effects of ferric (Fe^3+^) and ferrous (Fe^2+^) on osteoblast cell viability, proliferation and differentiation [20]. Similarly, previous studies from other groups also showed negative effects of iron on osteoblasts [9,58–61]. In this study, direct effects of Fe^3+^ and Fe^2+^ on the expression of osteoblast differentiation markers upon iron exposure were examined and compared. While both Fe^3+^ and Fe^2+^ did not significantly alter the expression of most osteoblast differentiation markers, both iron species markedly inhibited ALP expression (Fig 1). Similar to the previously reported data showing that Fe^3+^ had stronger negative effects on osteoblasts than Fe^2+^ [20], Fe^3+^ also showed the stronger suppressive effects on ALP expression in osteoblasts than Fe^2+^ in this study. These results also corresponded to the previous reports that both Fe^3+^ and Fe^2+^ could impair ALP activity [20,41,60,62,63]. A few studies also suggested the inhibitory roles of iron on the expression of osteoblast differentiation markers in C2C12 myoblast cells and human bone marrow multipotent mesenchymal stem cells (BMSCs) [9,64]. Accordingly, these results suggested that both iron species could have higher inhibitory effects on osteoblast differentiation markers in osteoblast precursor cells during osteogenic induction than in committed osteoblast cells. Interestingly, a slight induction of collagen 1A and OCN expression was observed in FAC treated groups (Fig 1E and 1G). Similar induction was observed in previous studies where OCN expression tended to increase in C2C12 cells treated with ferric sulfate [64]. Our results also showed that collagen type 1A mRNA expression was slightly increased in the presence of 300 μM FAC at 24 h, but this induction disappeared in the later time points (Fig 1E). This corresponded to previous studies showing that collagen I protein was down-regulated in osteoblasts treated with long-term exposure of FAC [41]. Future experiments can also be done to determine protein expression of these osteoblast differentiation markers. However, previous studies reported that the alterations of mRNA and protein expression of these osteoblast differentiation markers were well correlated in iron-treated osteoblasts and BMSCs [9,41]. ALP is an enzyme functioning in osteoblast mineralization by hydrolyzing inorganic pyrophosphate to phosphate for hydroxyapatite formation [65]. Hence, the effects of Fe^3+^ and Fe^2+^ on osteoblast mineralization from the measurement of calcium deposition in osteoblasts themselves and in peripheral extracellular matrices were further investigated. Our results also confirmed that FAC (Fe^3+^) inhibited osteoblast mineralization significantly and with the stronger degree than FAS (Fe^2+^). Although long-term exposure of certain iron concentration could also affect osteoblast viability, our previous published data [20] and results from the present study (Fig 1) showed that both Fe^2+^ and Fe^3+^ could suppress ALP activity and expression in UMR-106 cells. Therefore, a decreased ALP function may contribute to the negative effects of Fe^2+^ and Fe^3+^ on mineralization shown in this study.

In addition to direct effects of iron overload on bone cells that could aggravate osteoporosis, hypocalcemia and certain hormone deficiency may also contribute to osteoporosis. Interestingly, hypocalcemia and vitamin D deficiency have also been reported in thalassemia patients and animals, which could also contribute to thalassemia-induced osteoporosis [66,67]. Hypocalcemia was also evidenced in hemochromatosis [68]. Accordingly, vitamin D and/or calcium supplement may be treatment options for iron overload-induced osteoporosis. Previous study in our laboratory also demonstrated calcium absorption impairment in thalassemic mice. Moreover, administration of iron transport inhibitor, hepcidin or vitamin D_3_ could restore calcium transport in thalassemic mice [49]. These findings suggested that calcium transport had an inverse correlation with iron absorption in intestine. Our previous report also showed that both iron species could enhance intracellular iron in osteoblast exposed to FAC and FAS in a dose-dependent manner [20]. Moreover, similar set of iron transporters found in intestinal cells were also expressed in osteoblasts [20], but the direct effects of vitamin D on osteoblast iron uptake has never been elucidated. Therefore, effects of 1,25(OH)_2_D_3_ on intracellular iron of osteoblast and on osteoblast viability under iron overload with FAC and FAS were investigated. Our results showed that, surprisingly, 1,25(OH)_2_D_3_ significantly induced intracellular iron in osteoblasts treated with FAC and FAS (Fig 3). However, this increased intracellular iron in osteoblasts did not significantly alter osteoblast survival under FAC and FAS treatments (Fig 4). Since the reciprocal interaction between calcium and iron transport has been proposed in intestinal cells or in intestine of thalassemic mice, our report suggested that this phenomenon might not occur in osteoblasts. There is no previous report about the effects of 1,25(OH)_2_D_3_ on iron uptake in osteoblasts. However, previous study in human hepatocellular carcinoma reported that 1,25(OH)_2_D_3_ could mediate iron homeostasis in HepG2 cells and monocytes by suppressing hepcidin expression but promoting ferroportin and ferritin expression [69]. This report supported our hypothesis that 1,25(OH)_2_D_3_ could also have a non-calciotropic effects on osteoblasts leading to higher iron uptake capacity. Nevertheless, the mechanism behind 1,25(OH)_2_D_3_-induced intracellular iron in osteoblasts under iron overload still needs to be sought. Moreover, because the increased intracellular iron in osteoblasts treated with 1,25(OH)_2_D_3_ under iron overload did not significantly affect osteoblast cell viability (Fig 4), our results suggested that the level of intracellular iron did not directly correlate with osteoblast cell death under iron overload. To further verify this speculation, iron chelator, deferiprone (DFP) was selected to examine the effects on intracellular iron of osteoblasts and osteoblast viability under iron overload. Our results demonstrated that DFP effectively reduced intracellular iron in osteoblasts treated with FAC and FAS, especially under 1,25(OH)_2_D_3_ stimulation (Fig 5). Interestingly, DFP could not significantly rescue osteoblasts (Fig 6). These results suggested that DFP could not be use as a therapeutic or preventive agent for osteoblasts under iron overload, and the level of intracellular iron did not relate to osteoblast cell death under iron overload with FAC and FAS.

Hypocalcemia has been reported in iron overload patients as mentioned, and vitamin D_3_ did not show the promising results in osteoblast cell survival under iron overload (Fig 4). Therefore, calcium supplement could be another alternative to improve calcium homeostasis in thalassemia and iron overload patients. Accordingly, the direct effects of extracellular calcium treatment in a form of CaCl_2_ on osteoblast cell survival under iron overload has been tested in this study. Interestingly, the results showed that CaCl_2_ could effectively rescue osteoblasts from iron-induced osteoblast cell death (Fig 7). Even though the reciprocal interaction between calcium and iron transport has been suggested in intestine of thalassemia mice [49], our findings have already elaborated that this might not be the underlies rescue mechanism of iron-induced osteoblast cell death by CaCl_2_. This is because our results demonstrated that iron-induced osteoblast cell death was independent from the level of intracellular iron in osteoblasts (Figs 3, 4, 5 and 6). Instead, a few studies recently reported the beneficial effects of extracellular calcium on osteoblast cell proliferation and development [70,71]. Accordingly, the positive signals from extracellular calcium exposure on osteoblast cells might be able to counteract the negative signals from iron overload by FAC and FAS leading to the protective properties of extracellular calcium against iron-induced osteoblast cell death. Even if the mechanism behind the protective activity of CaCl_2_ was still unknown, extracellular calcium supplement or agents that could improve serum calcium level could be a strong candidate for the therapeutic agent for iron overload-induced osteoporosis, which could protect osteoblast cells from iron toxicity and improve hypocalcemia at the same time.

Several studies have reported that iron overload by FAC could lead to the increase of cellular reactive oxygen species (ROS) in osteoblasts leading to iron-induced osteoblast cell death [60,61,72,73]. Previous studies showed that increased ROS production could induce cell cycle arrest in osteoblasts [74,75]. However, there is no report whether Fe^2+^ could also induce ROS production in osteoblasts, and whether the two biological available iron species, Fe^3+^ and Fe^2+^, increased a comparable level of ROS production in osteoblasts. Accordingly, ROS production in UMR-106 osteoblast cells exposed to both Fe^3+^ and Fe^2+^ was investigated and compared. The results showed that both iron species could induce ROS production in UMR-106 cells with the stronger degree from Fe^3+^ (Fig 8). Moreover, Fe^3+^ and Fe^2+^ were found to induce G0/G1 cell cycle arrest in UMR-106 cells (Figs 9 and 10), consistent with the correlation between ROS production and G0/G1 cell cycle arrest as reported previously in MC3T3-E osteoblast-like cells [75]. Therefore, we speculated that both iron species used similar mechanism to induce osteoblast cell death via ROS production, and the use of chemical agents that could protect cells from ROS or inhibit ROS production could be another possible way to protect osteoblasts from iron-induced osteoblast cell death. More studies are needed to further elucidate this possibility.

In summary, this study provided crucial information about differential effects of two iron species, Fe^3+^ and Fe^2+^, on osteoblast cell differentiation, mineralization and ROS production as well as effects of potential therapeutic agents of osteoblast cell death under iron overload. In addition, effects of 1,25(OH)_2_D_3_ on iron uptake capacity by osteoblasts and the protective property of extracellular CaCl_2_ on osteoblast cell death under iron overload were firstly discovered. This study has provided the essential data for targeted therapeutic design for specific iron species contributing to osteoblast toxicity in patients with iron overload-induced osteoporosis. Last but not least we have shown that an increase in extracellular calcium by any mean may be beneficial for this group of patients.

## Supporting information

S1 Table *Rattus norvegicus* primers used in the qRT-PCR experiments. (DOCX)

S1 Fig Representative images of calcium mineralization by alizarin red staining of UMR-106 cells treated with FAC (A) and FAS (B). (EPS)
