# Supplementary material for: Differential effects of Fe2+ and Fe3+ on osteoblasts and the effects of 1,25(OH)2D3, deferiprone and extracellular calcium on osteoblast viability under iron-overloaded conditions
Source: PLoS One. 2020 May 29;15(5):e0234009. doi: 10.1371/journal.pone.0234009 (PMC7259719; doi:10.1371/journal.pone.0234009)
Supplement: S1 Table — (DOCX) [file pone.0234009.s001.docx]

**S1 Table***. Rattus norvegicus* primers used in the qRT-PCR experiments.

| Gene | Accession no. | Primer (forward/reverse) | Annealing temperature (°C) |
| --- | --- | --- | --- |
| ***Osteoblast differentiation markers*** | | | |
| Runt-related transcription factor 2 (Runx2) | NM_053470 | 5’–TAACGGTCTTCACAAATCCTC–3’  5’–GGCGGTCAGAGAACAAACTA–3’ | 54 |
| Collagen type 1A | NM_053304.1 | 5’–CAGTCGATTCACCTACAGCAC–3’  5’–GGGATGGAGGGAGTTTACACG–3’ | 59 |
| Alkaline phosphatase (ALP) | NM_013059 | 5’–AGAACTACATCCCCCACG–3’  5’–CAGGCACAGTGGTCAAGGT–3’ | 58 |
| Osteocalcin (OCN) | J04500 | 5’–CACAGGGAGGTGTGTGAG–3’  5’–TGTGCCGTCCATACTTTC–3’ | 57 |
| ***Housekeeping gene***  β-actin | NM_031144 | 5’–CAGAGCAAGAGAGGCATCCT–3’  5’–GTCATCTTTTCACGGTTGGC–3’ | 56 |
